# Immune Checkpoint Inhibitor Therapy in Patients With Preexisting Inflammatory Bowel Disease

**DOI:** 10.1200/JCO.19.01674

**Published:** 2019-12-04

**Authors:** Hamzah Abu-Sbeih, David M. Faleck, Biagio Ricciuti, Robin B. Mendelsohn, Abdul R. Naqash, Justine V. Cohen, Maclean C. Sellers, Aanika Balaji, Guy Ben-Betzalel, Ibraheim Hajir, Jiajia Zhang, Mark M. Awad, Giulia C. Leonardi, Douglas B. Johnson, David J. Pinato, Dwight H. Owen, Sarah A. Weiss, Giuseppe Lamberti, Mark P. Lythgoe, Lisa Manuzzi, Christina Arnold, Wei Qiao, Jarushka Naidoo, Gal Markel, Nick Powell, Sai-Ching J. Yeung, Elad Sharon, Michael Dougan, Yinghong Wang

**Affiliations:** ^1^ The University of Texas MD Anderson Cancer Center, Houston, TX; ^2^ Memorial Sloan Kettering Cancer Center, New York, NY; ^3^ Dana-Farber Cancer Institute, Boston, MA; ^4^ University of Perugia, Perugia, Italy; ^5^ East Carolina University, Greenville, NC; ^6^ Massachusetts General Hospital, Boston, MA; ^7^ Harvard Medical School, Boston, MA; ^8^ Johns Hopkins University, Baltimore, MD; ^9^ Sheba Medical Center, Tel Aviv, Israel; ^10^ Kings College London, London, United Kingdom; ^11^ University of Catania, Catania, Italy; ^12^ Vanderbilt University Medical Center, Nashville, TN; ^13^ Imperial College London, Hammersmith Hospital Campus, London, United Kingdom; ^14^ The Ohio State University, Columbus, OH; ^15^ Yale University, New Haven, CT; ^16^ Policlinico di Sant'Orsola University Hospital, Bologna University, Bologna, Italy; ^17^ National Cancer Institute, Bethesda, MD

## Abstract

**PURPOSE:**

The risk of immune checkpoint inhibitor therapy–related GI adverse events in patients with cancer and inflammatory bowel disease (IBD) has not been well described. We characterized GI adverse events in patients with underlying IBD who received immune checkpoint inhibitors.

**PATIENTS AND METHODS:**

We performed a multicenter, retrospective study of patients with documented IBD who received immune checkpoint inhibitor therapy between January 2010 and February 2019. Backward selection and multivariate logistic regression were conducted to assess risk of GI adverse events.

**RESULTS:**

Of the 102 included patients, 17 received therapy targeting cytotoxic T-lymphocyte antigen-4, and 85 received monotherapy targeting programmed cell death 1 or its ligand. Half of the patients had Crohn’s disease, and half had ulcerative colitis. The median time from last active IBD episode to immunotherapy initiation was 5 years (interquartile range, 3-12 years). Forty-three patients were not receiving treatment of IBD. GI adverse events occurred in 42 patients (41%) after a median of 62 days (interquartile range, 33-123 days), a rate higher than that among similar patients without underlying IBD who were treated at centers participating in the study (11%; *P* < .001). GI events among patients with IBD included grade 3 or 4 diarrhea in 21 patients (21%). Four patients experienced colonic perforation, 2 of whom required surgery. No GI adverse event–related deaths were recorded. Anti–cytotoxic T-lymphocyte antigen-4 therapy was associated with increased risk of GI adverse events on univariable but not multivariable analysis (odds ratio, 3.19; 95% CI, 1.8 to 9.48; *P* = .037; and odds ratio, 4.72; 95% CI, 0.95 to 23.53; *P* = .058, respectively).

**CONCLUSION:**

Preexisting IBD increases the risk of severe GI adverse events in patients treated with immune checkpoint inhibitors.

## INTRODUCTION

Immunotherapy targeting the immune checkpoint receptors cytotoxic T-lymphocyte antigen-4 (CTLA-4), programmed cell death 1 (PD-1), and programmed death ligand 1 (PD-L1) has proven effective in prolonging the survival of patients with a variety of advanced malignancies.^1-3^ Because CTLA-4 and PD-1/PD-L1 are fundamental regulators of immunity, such treatment can lead to a wide spectrum of inflammatory toxicities, collectively termed immune-related adverse events. These toxicities can involve any organ system, limit immunotherapy, and, in rare cases, be fatal.^4-6^ The molecular and cellular mechanisms driving immune-related adverse events are poorly understood, as are predisposing risk factors. Out of concern that patients with underlying autoimmune disease are at increased risk for developing severe immune-related adverse events, they have systematically been excluded from checkpoint inhibitor clinical trials.^5-7^

Inflammation of the small intestinal and colonic mucosa (immune-mediated enterocolitis) is one of the most common adverse events associated with CTLA-4 or PD-1/PD-L1 inhibition.^5,8,9^ Immune-mediated enterocolitis is a distinct clinical and pathologic entity but has many features resembling inflammatory bowel diseases (IBDs), such as ulcerative colitis and Crohn’s disease.^5,8,9^ The roles of CTLA-4 and PD-1/PD-L1 in IBD are unclear. CTLA-4 haploinsufficiency is associated with severe inflammation in the GI tract, among other organs,^10-12^ and polymorphisms in the *CTLA-4* gene have been linked to ulcerative colitis risk in Asian populations.^13^ PD-1 and PD-L1 are expressed by the colonic epithelium, and surface expression of PD-1/PD-L1 is higher in patients with IBD, suggesting a potential regulatory function.^14,15^

Several meta-analyses have suggested retrospectively that immune checkpoint inhibitors are generally safe in patients with low active or untreated autoimmune diseases treated with either the CTLA-4 inhibitor ipilimumab or PD-1/PD-L1 inhibitors.^16-19^ These reports are limited by heterogeneity among the autoimmune diseases reported.^16-19^ The risk of GI adverse events in patients with underlying IBD who receive immunotherapy has been low in the few published studies^16-20^; however, with small numbers of patients and insufficient clinical characterization of the underlying IBD, the generalizability of these findings is limited, especially in patients with more active IBD. Because patients with IBD are at increased risk of several malignancies that are indications for immunotherapy, understanding how immunotherapy affects patients with IBD is critical and may further elucidate the roles of these immune regulatory pathways in IBD.^21-24^

## PATIENTS AND METHODS

### Patient Population

We performed an international, multicenter, retrospective cohort study of patients with cancer and underlying IBD who received immune checkpoint inhibitor therapy between January 2010 and February 2019. Appendix Table A1 (online only) lists the participating centers. Approval was obtained from the participating institutions’ institutional review boards. Thereafter, a universal data collection protocol was used among all centers to facilitate congruence of collected variables. Patients were included only if they had clear documentation of underlying IBD (ie, proven histologically or treated medically with IBD-specific therapy). A search for eligible patients using institutional databases (eg, pharmacy, gastroenterology clinic, oncology clinic, and investigational new drugs) and tumor registries was completed, followed by a comprehensive chart review. To compare the rate of GI adverse events, we included a control cohort of patients without underlying IBD who received immune checkpoint inhibitor therapy at some of the participating institutions (Appendix Table A1) and documented the type of immunotherapy received and the rate of GI adverse events. Determination of presence and grade of GI adverse events in the control group was done in a similar fashion to that of patients with IBD; institutional databases were searched, followed by thorough chart review to confirm the diagnosis.

### Clinical Characteristics

Clinical data consisted of demographics, oncologic and medical history, and Charlson comorbidity index score.^25^ These data were extrapolated retrospectively from medical charts. Variables related to oncologic history included cancer type and stage at the time of immunotherapy initiation, immune checkpoint inhibitor type, treatment duration, and non-GI immune-related adverse events.

### IBD Assessment

IBD was classified as Crohn’s disease, ulcerative colitis, or unclassified. The time between IBD diagnosis or last active IBD episode and immunotherapy initiation was reported. IBD treatment within 3 months before immunotherapy initiation was categorized as mesalamine, immunosuppressive (eg, glucocorticoids, infliximab, vedolizumab, ustekinumab, adalimumab, azathioprine, mercaptopurine), or none. Findings of the last available endoscopic evaluation were noted, and endoscopic presentation was categorized as mild, moderate, or severe in a similar fashion as that used for ulcerative colitis (ie, using the Mayo score in patients with ulcerative colitis and based on the clinician impression in patients with Crohn’s disease).^26^ We also documented the presence of IBD-related complications (ie, colonic stricture, fistula, perforation, GI cancer), surgical interventions, or extraintestinal manifestations before immunotherapy, as well as the distribution of IBD in the GI tract.

### GI Adverse Events

Recorded GI adverse events consisted of diarrhea, colitis, nausea, and vomiting. We recorded time from immunotherapy initiation to GI symptom onset (if any); peak grade of diarrhea and colitis according to the Common Terminology Criteria for Adverse Events version 5.0^27^; requirement for hospitalization or intensive care unit admission; treatment of GI adverse events; cumulative duration of symptoms, hospitalization, and corticosteroid treatment; endoscopic presentation; colonic perforation with or without surgical intervention; recurrence of symptoms; and time from resolution of first episode to recurrence.

### Statistical Analysis

A descriptive summary of continuous variables using medians and interquartile ranges (IQRs) and of categorical variables using frequencies and percentages was performed. Fisher’s exact test was used to compare categorical variables, and the Wilcoxon rank sum test was used to compare continuous variables. A univariable logistic regression analysis was conducted to assess for risk factors of GI adverse events. Multivariable analyses with backward model selection were performed to assess for independent risk factors for GI adverse events. The Hosmer-Lemeshow test was used to check the goodness of fit for the final model, with a *P* = .14 indicating good fit. Statistical tests were 2-sided. A *P* = .05 was used as a threshold for statistical significance. Statistical analyses were performed using SPSS version 24.0 (SPSS, Chicago, IL) and SAS version 9.4 (SAS Institute, Cary, NC).

## RESULTS

### Patients

A total of 102 patients with cancer and IBD received immunotherapy with CTLA-4 or PD-1/PD-L1 inhibitors and were included in the study. Patient characteristics are listed in Table 1. Most patients (83%) received PD-1/PD-L1 inhibitor monotherapy.

**TABLE 1.**
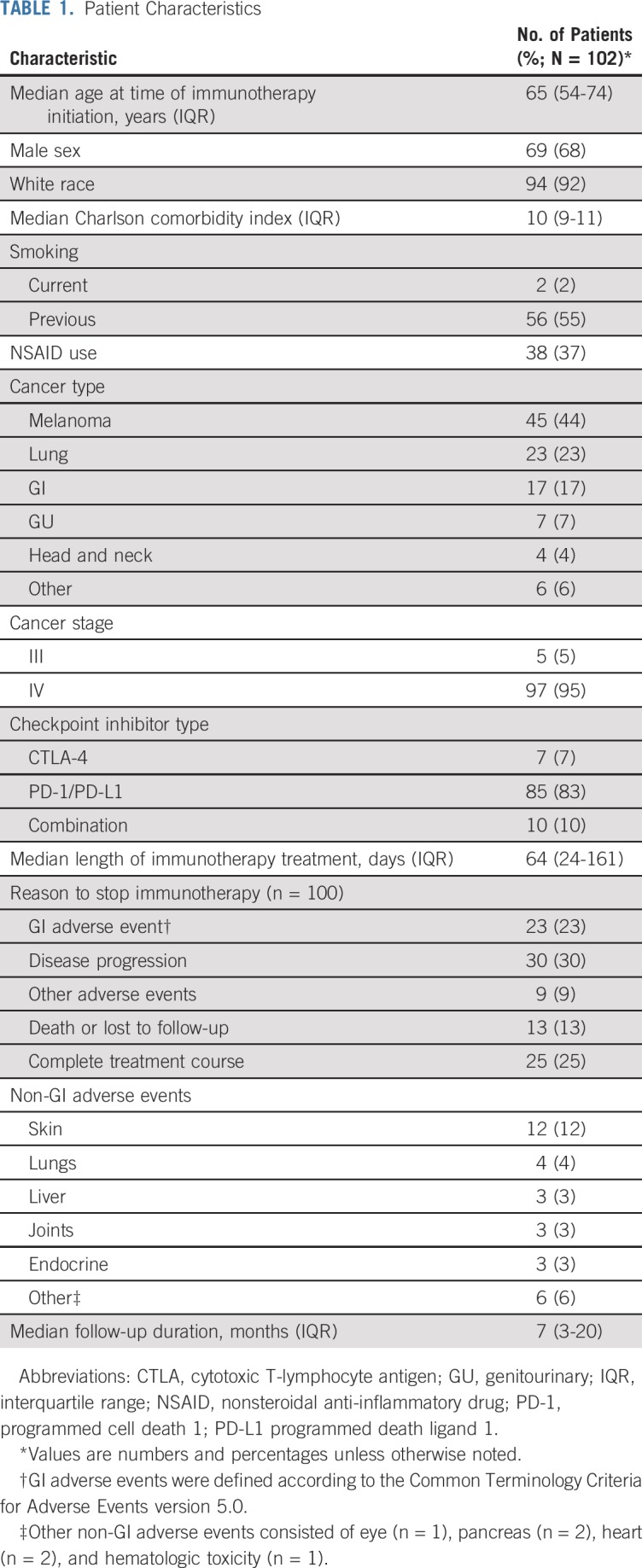
Patient Characteristics

### Underlying IBD

The study included 49 patients with Crohn’s disease, 49 with ulcerative colitis, and 4 with unclassified IBD (Table 2). The median time from last active IBD episode to immunotherapy initiation was 5 years (IQR, 3-12 years). Forty-two percent of patients had not received IBD treatment within the 3 months before immunotherapy (Appendix Table A2, online only). Most patients with available endoscopy data before immunotherapy had normal findings (29 of 48 patients; 60%) on the most recent endoscopy before immunotherapy, with a median time from endoscopy to immunotherapy of 17 months.

**TABLE 2.**
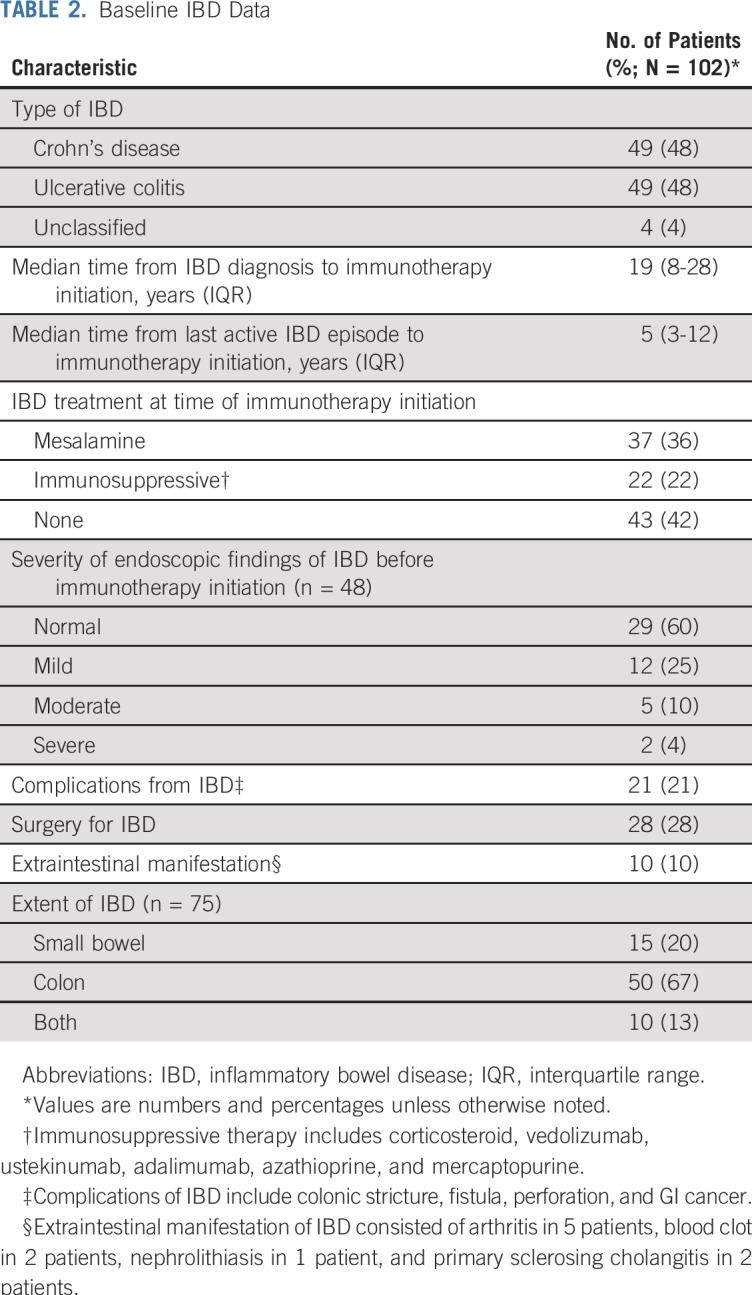
Baseline IBD Data

### GI Adverse Events

**FIG A1.**
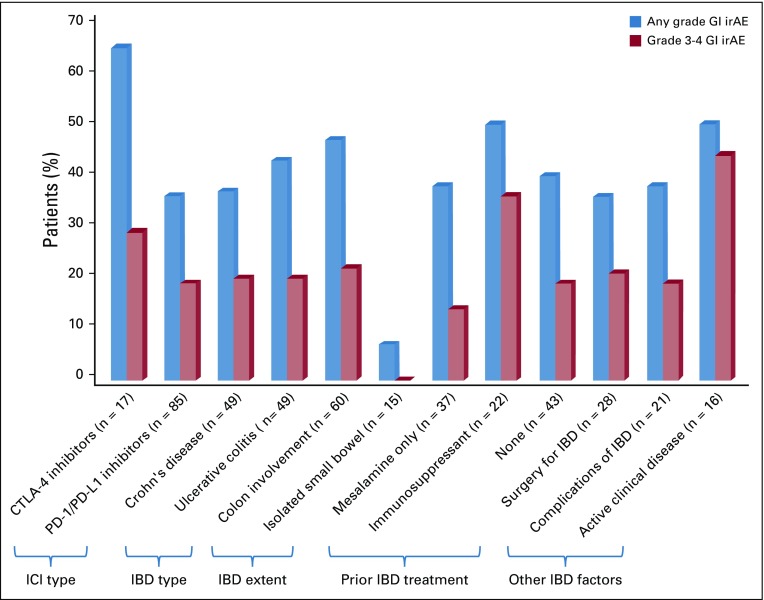
Bar graph of GI immune-related adverse events (irAEs) among different subgroups. CTLA-4, cytotoxic T-lymphocyte antigen-4; IBD, inflammatory bowel disease; ICI, immune checkpoint inhibitor; PD-1, programmed cell death 1; PD-L1 programmed death ligand 1.

Of the 102 patients, 42 (41%) had a GI adverse event after a median of 62 days (IQR, 33-123 days) after immunotherapy initiation (Table 3; Appendix Fig A1, online only), and 23 of these patients stopped immunotherapy because of GI adverse events. Of the 41 patients who experienced diarrhea, half (51%) had a peak grade of diarrhea of 3 or 4. Most patients (76%) received glucocorticoids, with 29% requiring treatment escalation to include infliximab or vedolizumab. One patient received adalimumab and 1 patient received mesalamine for a GI adverse event. Of the 32 patients who underwent endoscopy for adverse events, 38% had mucosal ulceration and 44% had nonulcerative inflammation.

**TABLE 3.**
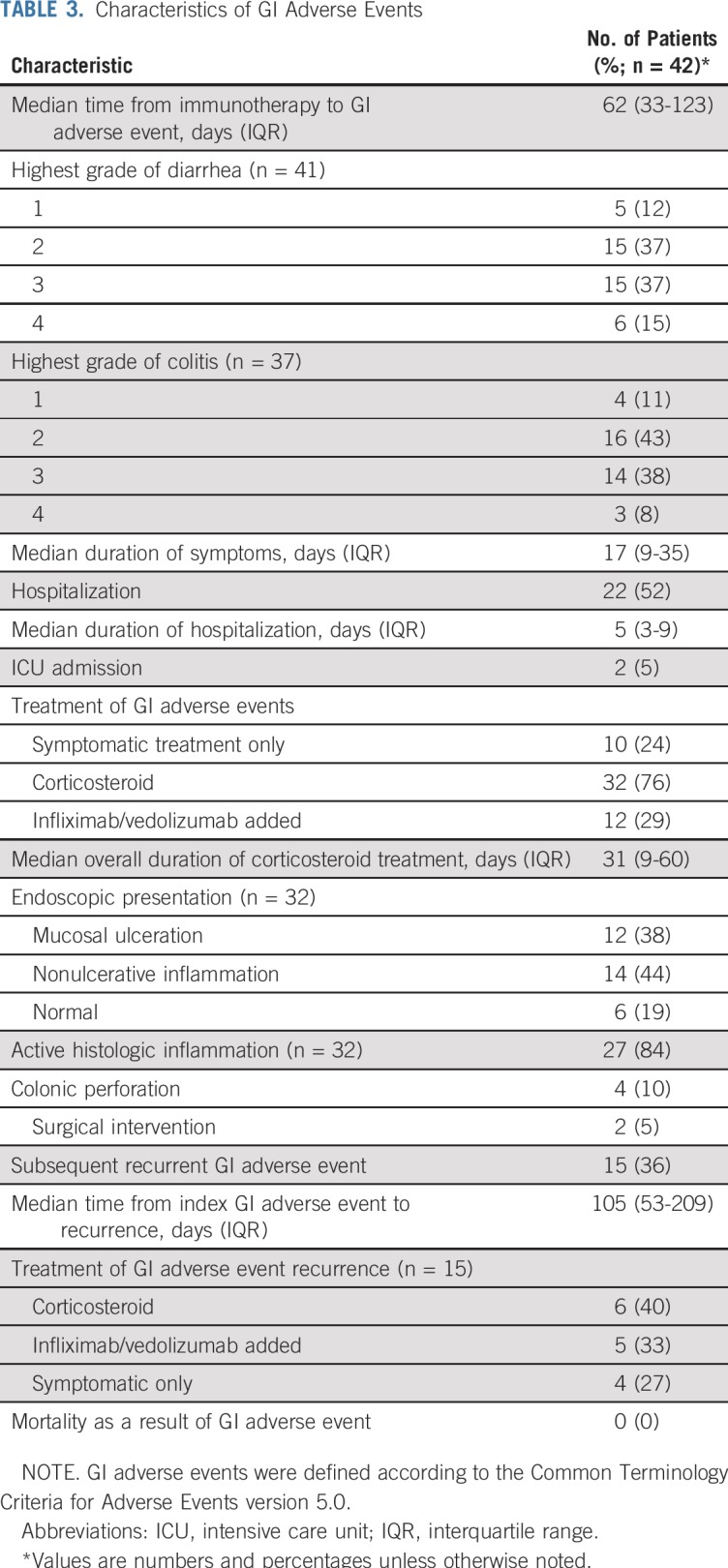
Characteristics of GI Adverse Events

In patients without IBD, the overall rate of GI adverse events of any grade was 11% (1,283 patients had a GI adverse event of the 11,377 patients who received either CTLA-4 [18%] or PD-1/PD-L1 [82%] inhibitors). The proportion of GI adverse events of any grade in patients with preexisting IBD was significantly higher than that of patients without underlying IBD (41% *v* 11%, respectively; *P* < .001).

Colonic perforation occurred in 4 patients (2 with ulcerative colitis and 2 with Crohn’s disease); 2 of these patients were treated conservatively, and 2 underwent surgery. The patients who experienced colonic perforation received glucocorticoids without infliximab or vedolizumab before the perforation. Of these 4 patients, 3 had received anti–PD-1/PD-L1 monotherapy and 1 had received combined anti–CTLA-4 and anti–PD-1/PD-L1 therapy.

Among the 42 patients who experienced adverse events, 15 experienced an additional episode of GI symptoms after a median of 105 days (IQR, 53-209 days) from resolution of the first episode. No GI toxicity–related deaths were recorded in the study.

**FIG A2.**
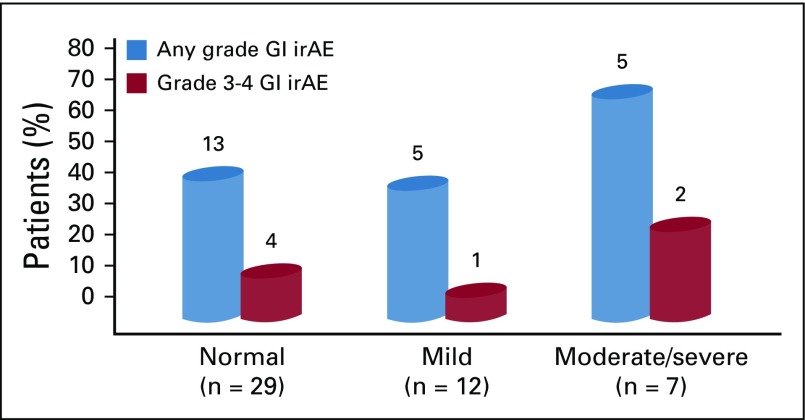
Bar graph of GI immune-related adverse events (irAEs) according to endoscopic presentation at time of immune checkpoint inhibitor initiation.

Rate and grade of GI adverse events according to endoscopic presentation of IBD at time of immune checkpoint inhibitor initiation are shown in Appendix Figure A2 (online only). Of the 28 patients who had undergone surgical intervention for IBD before immunotherapy, 25% had GI adverse events. Of the 15 patients who had isolated small bowel involvement, only 1 patient developed a GI adverse event.

In univariable analysis, anti–CTLA-4 therapy (either monotherapy or combination therapy) was associated with a higher risk of GI adverse events compared with anti–PD-1/PD-L1 therapy (odds ratio, 3.19; 95% CI, 1.8 to 9.48; *P* = .037). We did not observe any other factors with a significant association (Table 4). In multivariable logistic regression, none of the variables reached statistical significance. Nonetheless, anti–CTLA-4 therapy (odds ratio, 4.72; 95% CI, 0.95 to 23.53; *P* = .058) and IBD involving the colon (odds ratio, 3.61; 95% CI, 0.85 to 15.27; *P* = .081) showed tendency for an increased risk of GI adverse events (Table 5). Patients who had active IBD within 3 months before immunotherapy initiation had higher grades of diarrhea compared with patients with inactive IBD (*P* = .027; Appendix Table A3, online only). No significant differences in clinical severity of GI adverse events were observed between patients who received IBD treatment at time of immune checkpoint inhibitor initiation and those who did not (Appendix Table A4, online only).

**TABLE 4.**
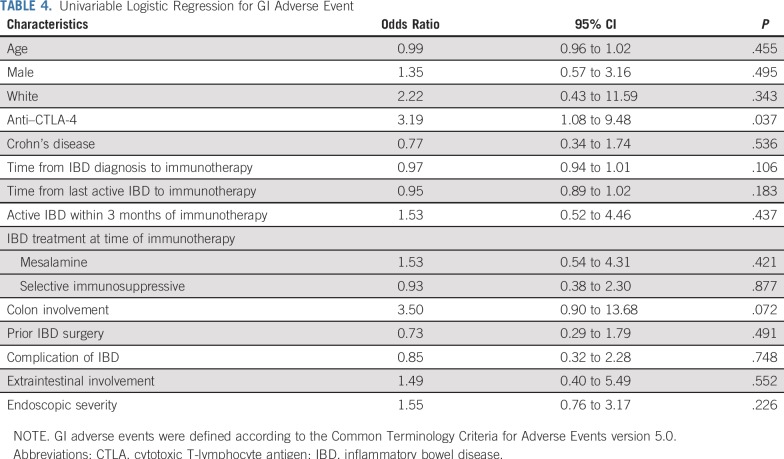
Univariable Logistic Regression for GI Adverse Event

**TABLE 5.**
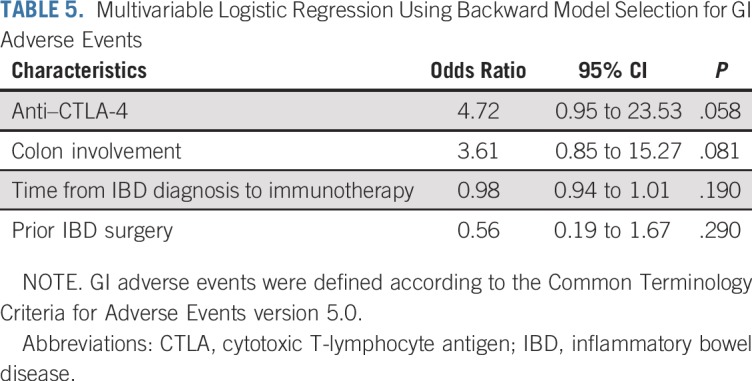
Multivariable Logistic Regression Using Backward Model Selection for GI Adverse Events

### Cancer Outcome

In this cohort, 53 patients (52%) had progressive disease at the end of immune checkpoint inhibitor therapy; the rest had either stable disease or complete or partial response to immunotherapy.

## DISCUSSION

This is the largest multicenter study to date examining the risk of GI adverse events in patients with cancer and underlying IBD who received immunotherapy with CTLA-4 or PD-1/PD-L1 inhibitors. We observed a 41% rate of GI adverse events in the study cohort, which was considerably higher than the expected incidence of GI adverse events in patients without preexisting IBD and higher than that observed in a control cohort without IBD. In addition, patients with preexisting IBD had a high rate of severe GI adverse events (ie, high grades of diarrhea and colitis, frequent requirement for infliximab or vedolizumab, high rates of colonic perforation requiring surgical intervention, and frequent recurrence). Nonetheless, no deaths caused by GI toxicity were recorded. The clinical benefit rate of immune checkpoint inhibitor therapy was similar to that reported in clinical trials.^28-31^

Our findings differ from previous reports of immunotherapy in patients with underlying IBD. Prior retrospective analyses evaluating patients with underlying autoimmune diseases included small numbers of patients with IBD.^16-18^ In addition, minimal data on IBD histories and prior diagnostic evaluations were provided. We have provided substantially more clinical details on IBD and GI adverse events than those previously reported, thereby substantiating the diagnosis of IBD, reducing the impact of selection bias, and facilitating the application of our results to other clinical situations.

In our study, anti–CTLA-4 therapy was not associated with a significantly increased risk of GI adverse events compared with anti–PD-1/PD-L1 therapy; however, this association was significant in the univariable analysis and showed a tendency toward significance in the multivariable analysis. The lack of significance of this finding may result from the small number of patients receiving anti–CTLA-4 therapy in our cohort (17%), potentially because of concerns about the risk of using CTLA-4 inhibitors in this population. However, we cannot exclude the possibility that both CTLA-4 and PD-1/PD-L1 play similar key roles in IBD regulation, although mucosal homeostasis is largely controlled by CTLA-4 in the healthy gut.^5^ Johnson et al^16^ reported that ipilimumab was associated with a moderate risk for GI adverse events, with 2 of 6 patients having an IBD flare. PD-1 inhibitors were not associated with GI adverse events in any of the 12 patients with IBD from 2 retrospective cohorts.^17,18^

Although no fatalities were reported, the rate of colonic perforation in the current cohort is higher than the published risk of colonic perforation in patients with immune-mediated enterocolitis (approximately 2%).^32^ This finding underscores the importance of monitoring patients with IBD on immunotherapy, who are at increased risk of serious GI complications, to allow for early identification and initiation of therapy for GI adverse events. Moreover, thorough evaluation of patients with IBD who develop a suspected GI toxicity is critical to ensure appropriate management. Even in patients without IBD, endoscopic and histologic assessment can help guide appropriate management.^9,32^ Early introduction of infliximab or vedolizumab is associated with improved outcomes in immune-mediated enterocolitis, principally in patients with severe endoscopic and histologic presentation.^33^ Although rare, immune-mediated enterocolitis that is refractory to immunosuppressive therapy may respond to fecal microbiota transplantation.^34^

The rate of GI adverse events was high in the current study, yet the benefits of immunotherapy in this population likely outweighed the risks. We observed no GI-related deaths, and the overall response rate in our cohort was high and consistent with published response rates for immunotherapy. Future studies with long-term follow-up controlling for tumor type and other covariates are needed to assess cancer response in patients with IBD who experience GI adverse events.

Although we found that active IBD within 3 months before immune checkpoint inhibitor therapy was not associated with significant risk of GI adverse events, patients with active IBD had more severe GI adverse events. Therefore, appropriate treatment of the underlying IBD before immunotherapy initiation may be beneficial, optimally with endoscopic confirmation of mucosal healing. However, whether to withhold immunotherapy until IBD remission in a patient with active cancer remains an open question. In a recent report, the resumption of immune checkpoint inhibitor therapy after the resolution of immune-related GI adverse events was associated with a 33% risk of adverse event recurrence, suggesting a pattern similar to IBD.^35^

The effect of concurrent IBD therapy alongside immunotherapy could not be delineated in the current analysis because of the heterogeneity of treatments and study size; this approach is worth additional investigation in a prospective effort. Similarly, whether surgical removal of the involved part of the GI tract is protective against GI adverse events could not be definitively established here, although we found that the rate of GI adverse events was numerically lower (25%) in such patients. Although the numbers were limited, 5 of the 7 patients with moderate to severe endoscopic findings before immunotherapy initiation had GI adverse events. Future prospective studies should assess the utility of endoscopic evaluation in the decision to initiate immune checkpoint inhibitor therapy in patients with preexisting IBD.

The study has several limitations. First, the retrospective nature could have led to incomplete data collection, particularly of medication- and endoscopy-related variables, because many patients’ gastroenterologic care was provided outside of the cancer centers in this study. Second, because IBD was an exclusion criterion for most clinical trials involving immunotherapy, the decision to give immune checkpoint inhibitor therapy was at the discretion of the treating oncologist and was not standardized. Therefore, the rate of GI adverse events could have been higher if patients with severe, active IBD were given immunotherapy, because the majority of patients in our cohort had inactive disease at the time of treatment. Fewer patients in our cohort were receiving IBD therapy (58%) compared with patients nationally (approximately 75%), indicative of selection for patients with mild disease.^36^ Third, given the macroscopic and microscopic similarities between immune-mediated enterocolitis and an IBD flare, these occurrences could not be distinguished from each other. Fourth, although having data from multiple institutions allows for generalizability, treatment standards differed among centers, especially in the absence of guidelines, which could have affected the outcomes of GI adverse events. Fifth, despite being the largest cohort to date, our sample size limited our ability to perform advanced and subgroup analyses. Last, our cohort comprised patients with various cancer types, precluding definitive cancer outcome analyses.

In conclusion, our findings suggest a role for both CTLA-4 and PD-1/PD-L1 in the pathogenesis of IBD and indicate that preexisting IBD increases the risk of severe GI adverse events in patients treated with immune checkpoint inhibitors. Specifically, anti–CTLA-4 therapy and IBD involving the colon before immunotherapy initiation were possible risk factors for GI toxicities. Nonetheless, response to immune checkpoint inhibitor therapy in patients with underlying IBD is comparable to what is reported in non-IBD patients, emphasizing the potential clinical benefit. Future large-scale prospective studies are warranted to clarify our findings.

## Figures and Tables

**TABLE A1.**
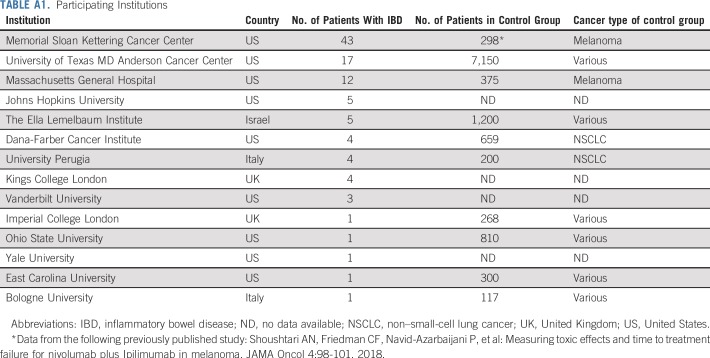
Participating Institutions

**TABLE A2.**
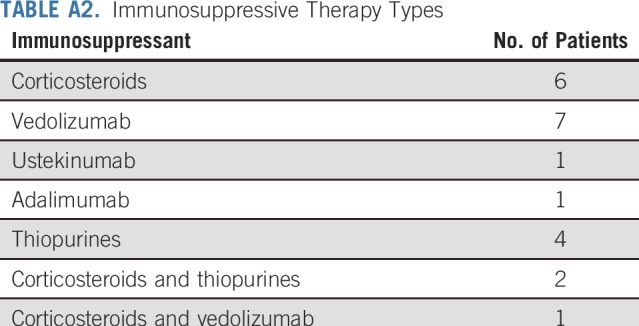
Immunosuppressive Therapy Types

**TABLE A3.**
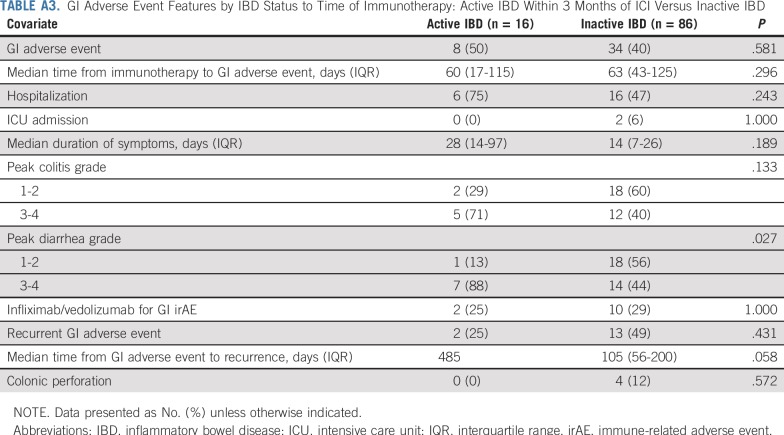
GI Adverse Event Features by IBD Status to Time of Immunotherapy: Active IBD Within 3 Months of ICI Versus Inactive IBD

**TABLE A4.**
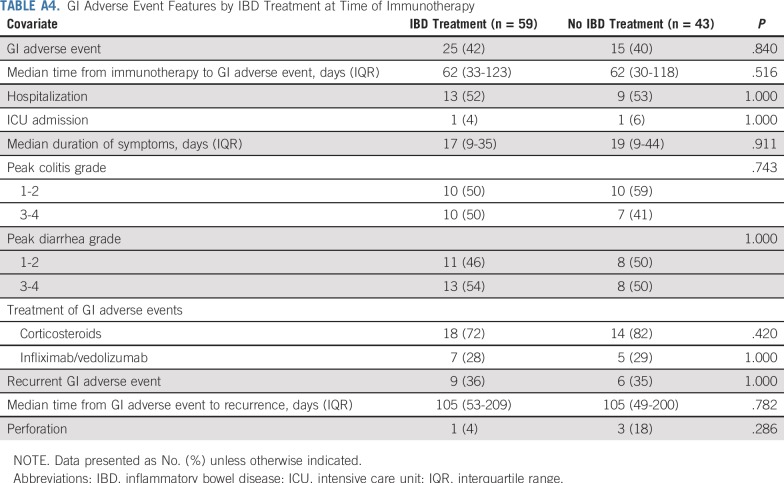
GI Adverse Event Features by IBD Treatment at Time of Immunotherapy
